# PLGA, PLGA-TMC and TMC-TPP Nanoparticles Differentially Modulate the Outcome of Nasal Vaccination by Inducing Tolerance or Enhancing Humoral Immunity

**DOI:** 10.1371/journal.pone.0026684

**Published:** 2011-11-02

**Authors:** Chantal Keijzer, Bram Slütter, Ruurd van der Zee, Wim Jiskoot, Willem van Eden, Femke Broere

**Affiliations:** 1 Department of Infectious Diseases and Immunology, Utrecht University, Utrecht, The Netherlands; 2 Division of Drug Delivery Technology, Leiden/Amsterdam Center for Drug Research (LACDR), Leiden, The Netherlands; National Institute of Environmental Health Sciences, United States of America

## Abstract

Development of vaccines in autoimmune diseases has received wide attention over the last decade. However, many vaccines showed limited clinical efficacy. To enhance vaccine efficacy in infectious diseases, biocompatible and biodegradable polymeric nanoparticles have gained interest as antigen delivery systems. We investigated in mice whether antigen-encapsulated PLGA (poly-lactic-co-glycolic acid), PLGA-TMC (N-trimethyl chitosan) or TMC-TPP (tri-polyphosphate) nanoparticles can also be used to modulate the immunological outcome after nasal vaccination. These three nanoparticles enhanced the antigen presentation by dendritic cells, as shown by increased *in vitro* and *in vivo* CD4^+^ T-cell proliferation. However, only nasal PLGA nanoparticles were found to induce an immunoregulatory response as shown by enhanced Foxp3 expression in the nasopharynx associated lymphoid tissue and cervical lymph nodes. Nasal administration of OVA-containing PLGA particle resulted in functional suppression of an OVA-specific Th-1 mediated delayed-type hypersensitivity reaction, while TMC-TPP nanoparticles induced humoral immunity, which coincided with the enhanced generation of OVA-specific B-cells in the cervical lymph nodes. Intranasal treatment with Hsp70-mB29a peptide-loaded PLGA nanoparticles suppressed proteoglycan-induced arthritis, leading to a significant reduction of disease. We have uncovered a role for PLGA nanoparticles to enhance CD4^+^ T-cell mediated immunomodulation after nasal application. The exploitation of this differential regulation of nanoparticles to modulate nasal immune responses can lead to innovative vaccine development for prophylactic or therapeutic vaccination in infectious or autoimmune diseases.

## Introduction

Nasal vaccination is described for the prevention of infectious diseases such as hepatitis B [1], [2] or influenza [3], [4]. However, recently, nasal antigen application has also become of interest as a route of vaccination in the field of autoimmunity [5]–[9] and allergy [10], [11]. Similar to other forms of mucosal immunization, nasal antigen application can stimulate antigen-specific responses locally and in the peripheral mucosal tissues [12]–[15]. Vaccination via the nasal mucosa might be preferred over oral vaccination given the low proteolytic activity in the nasal mucosa; this route of immunization requires a lower dose of antigen than that of oral immunization, which might also reduce the change of producing negative side-effects [16]. The immune response induced following mucosal antigen application depends on many factors, such as the nature of the antigen (soluble versus particulate), antigen dose, size and delivery to the mucosal tissues [16]. Although the immune response that is induced following mucosal antigen application depends on the antigen that is used, nanoparticle characteristics might also play an important role. For tolerance induction to self antigens used for vaccination in autoimmune diseases one would prefer to combine a self antigen with a vaccine that favors tolerance induction. On the other hand, in the case of prevention of infectious diseases an immunogenic antigen with a vaccine that enhances humoral immunity is preferred. Therefore, rational future vaccine design might benefit from knowledge of immunomodulatory characteristics of nanoparticles. In recent years, several *in vivo* studies have been conducted to investigate nanoparticle-mediated delivery of antigen at mucosal sites. Nanoparticles are available as non-toxic delivery systems with promise for nasal vaccination [17]–[20]. Since mucosal antigen application elicits different immune responses such as T-helper 2 (TH2)-mediated humoral immunity or T-helper 1 (TH1)-mediated Delayed-Type Hypersensitivity (DTH) [21]–[23], we explored if nanoparticles can differentially modulate the outcome of nasal vaccination. The readout to evaluate the efficacy of the applied vaccine relies mostly on induction of humoral immune responses as indicated by increased antigen-specific antibody titers [4], [18], [24]. However, this does not give insight into the underlying immunological mechanisms that drive the response towards humoral immunity or DTH. Marazuela et al. previously showed that intranasal administration of PLGA particles that contained a peptide with the major T-cell epitope of Ole e 1 induced a modified Th2 response and prevented mice from allergic sensitization of the whole protein [25], [26]. However, little is still known about the role of CD4^+^ T-cells in nasal vaccination and how different nanoparticle treatment might influence the activation of these cells, locally and in the peripheral tissues.

To study the mechanisms behind humoral immunity or DTH after nasal vaccination, three polymeric nanoparticles were analyzed that are efficient antigen delivery systems; PLGA (poly-lactic-co-glycolic acid), PLGA-TMC (N-trimethyl chitosan) and TMC-TPP (tri-polyphosphate) nanoparticles that all contained the model antigen ovalbumin (OVA). These particles have a similar average diameter of 250–500 nm, but differ in their surface charge and antigen release kinetics [17], [18].

For example, nasal application of TMC-TPP particles has been described in the field of influenza vaccination and elicits humoral immune responses as shown by a significant increase in antigen-specific IgG1/IgG2a serum titers and increased sIgA titers in nasal washes [4]. In contrast to induction of humoral immunity, mice fed a single dose of 40 µg of type II collagen (CII)-containing PLGA particles had reduced severity of arthritis and reduced anti-CII-specific IgG antibody titers and CII-specific T-cell responses [24].

We investigated in an OVA-specific DTH model whether nanoparticles itself cause a shift in the immunological outcome after nasal antigen application as shown by the activation and differentiation of CD4^+^ T-cell responses both at the site of vaccination and systemically. In addition, we investigated the relevance of nanoparticle mediated mucosal tolerance after nasal application in a proteoglycan induced arthritis (PGIA) model [27] with heat shock protein 70 (Hsp70) peptide mB29a encapsulated nanoparticles. Hsp70 is known to be one of the most conserved Hsps and has been shown to have disease suppressive properties [28]–[30]. Mouse Hsp70-peptide mB29a has been shown to be immunosuppressive after nasal application in the PGIA model [31]. Therefore, we explored the additional immunosuppressive effect of nanoparticle treatment by encapsulating the mB29a peptide.

This study was performed to obtain more insight into the mechanism by which nanoparticles drive the immune response towards immunomodulatory responses. The data may assist future rational vaccine design for prophylactic or therapeutic vaccination in infectious and autoimmune diseases, respectively.

## Results

### Differential uptake of FITC labeled OVA by BMDCs after nanoparticle treatment

We previously showed nanoparticle differences in association with and uptake by DCs as visualized by tracing uptake of OVA *in vitro* [17], [18]. These differences in association might modulate the subsequent antigen presenting capacity of DCs.

To investigate this, we treated DCs *in vitro* with OVA encapsulated in PLGA, PLGA-TMC or TMC-TPP nanoparticles or soluble OVA (sOVA) as a control and studied phenotypic and functional differences between treated DCs. No differences in DC maturation or viability were observed as analyzed by CD11c, MHC-class-II, CD40, CD86 and 7-AAD staining after nanoparticle treatment at OVA concentrations varying from 1 ng/ml to 1 µg/ml. We did not detect significant differences in cytokine profiles in culture supernatants (data not shown).

**Figure 1:**
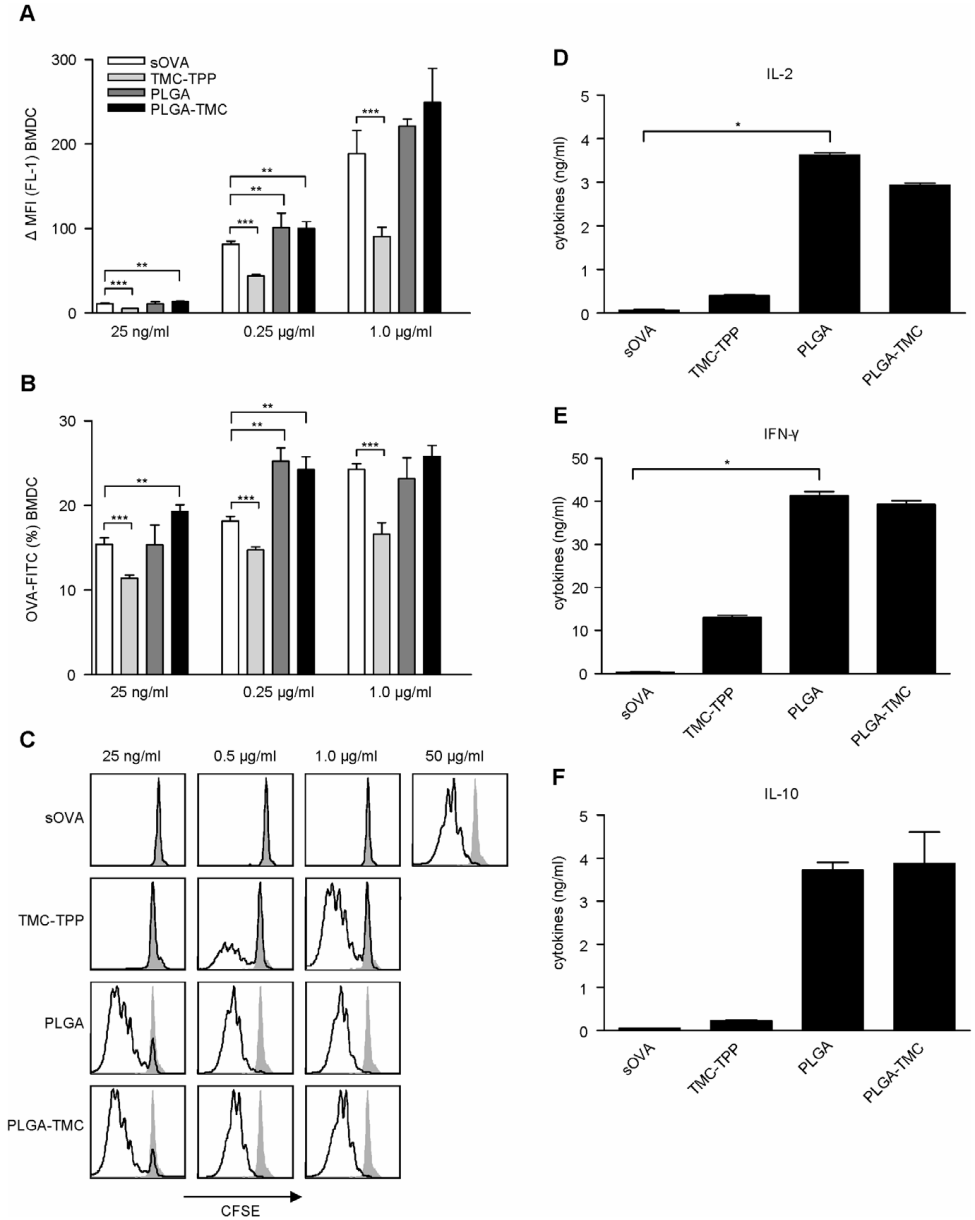
Nanoparticle mediated enhanced antigen presentation capacity of BMDCs *in vitro*. BMDC were incubated in the presence of sOVA-FITC or OVA-FITC encapsulated into PLGA, PLGA-TMC or TMC-TPP nanoparticles at different concentrations. External FITC signaling was silenced by trypan blue. **A.** The ΔMFI of OVA-FITC was assessed by subtraction of FITC signaling at 4°C from 37°C. **B.** OVA-FITC uptake by BMDC shown as the net percentage of OVA-FITC positive cells. **C.** CFSE-labeled CD4^+^ T-cells were incubated with BMDC stimulated with sOVA or OVA encapsulated in nanoparticles. Gray filled histograms; unstimulated CD4^+^ T-cells, Black overlays; CD4^+^ T-cell division patterns at different OVA concentrations after 72 hours. **D–F.** Cytokine concentrations of IL-2, IFN-γ and IL-10 (ng/ml) were determined in culture supernatants, after 72 h of culture. Data are representative for 3 independent experiments; mean ± SEM. Statistically significant: *, *P*<0.05; **, *P*<0.01.

Next, we studied the uptake of OVA-FITC encapsulated in PLGA, PLGA-TMC and TMC-TPP nanoparticles by DCs silencing extracellular attached FITC signaling with trypan blue. OVA-FITC association with DCs is shown as the FITC expression (ΔMFI) (Figure 1A) or percentage of OVA-FITC positive cells (Figure 1B).

OVA-FITC uptake by DCs treated with TMC-TPP was lower compared to sOVA-FITC treatment as shown by a low FITC ΔMFI expression and decreased percentage of OVA-FITC positive cells (Figures 1A and 1B). Furthermore, compared to sOVA, PLGA-TMC treatment enhanced the antigen uptake by DCs even at low (25 ng/ml) OVA concentrations. Both PLGA and PLGA-TMC treatment enhanced antigen uptake with OVA at 0.25 µg/ml. Apart from a reduced uptake of TMC-TPP particles we could not detect differences in antigen uptake at 1 µg/ml between sOVA, PLGA or PLGA-TMC treatment. The latter is being suggestive of saturated antigen uptake after 1.5 h of antigen incubation (Figure 1B).

In sum, nanoparticle characteristics differentially affected the antigen uptake by DCs *in vitro* as shown by a lower number of OVA-FITC positive cells and ΔMFI when DCs encounter TMC-TPP particles compared to PLGA and PLGA-TMC.

### Nanoparticles enhance OVA-specific CD4^+^ T-cell proliferation *in vitro*

To investigate whether the small differences in antigen uptake by DCs could affect the antigen presenting capacity of DCs, nanoparticle treated DCs were studied *in vitro* by co-culture with OVA-specific T-cells. DCs treated with OVA encapsulated PLGA, PLGA-TMC or TMC-TPP particles were cultured for 72 h in the presence of OVA-specific CFSE-labeled CD4^+^ T-cells.

The antigen presenting capacity of DCs was enhanced after nanoparticle treatment since T-cells stimulated by particle treated DCs showed enhanced T-cell proliferation compared to T-cells cultured in the presence of sOVA treated DCs. Especially, PLGA and PLGA-TMC particles strongly enhanced CD4^+^ T-cell proliferation even at a low OVA concentration of 25 ng/ml (Figure 1C). Additionally, in the culture supernatants of T-cells stimulated in the presence of 1 µg/ml of OVA containing PLGA or PLGA-TMC particles more IL-2 (Figure 1D), IFN-γ (Figure 1E) and IL-10 (Figure 1F) was detected compared to cultures with TMC-TPP particles or sOVA.

No T-cell proliferation or cytokine secretion was induced by empty nanoparticles (data not shown).

In conclusion, all three OVA loaded nanoparticles enhanced the antigen presentation by DCs, as shown by increased CD4^+^ T-cell proliferation profiles as compared to sOVA.

### Nasal vaccination enhances *in vivo* CD4^+^ T-cell activation and differentially induces Foxp3 expression

Next, we questioned whether nanoparticle treatment also affected CD4^+^ T-cell responses *in vivo*. Previously, we showed that especially TMC-TPP nanoparticles enhanced generation of antigen-specific IgG1 and IgG2a antibody titers after both i.n. and i.m. vaccination, whereas PLGA and PLGA-TMC only resulted in higher IgG titers after i.m vaccination and had little effect on the humoral immune response after i.n. treatment [18].

**Figure 2:**
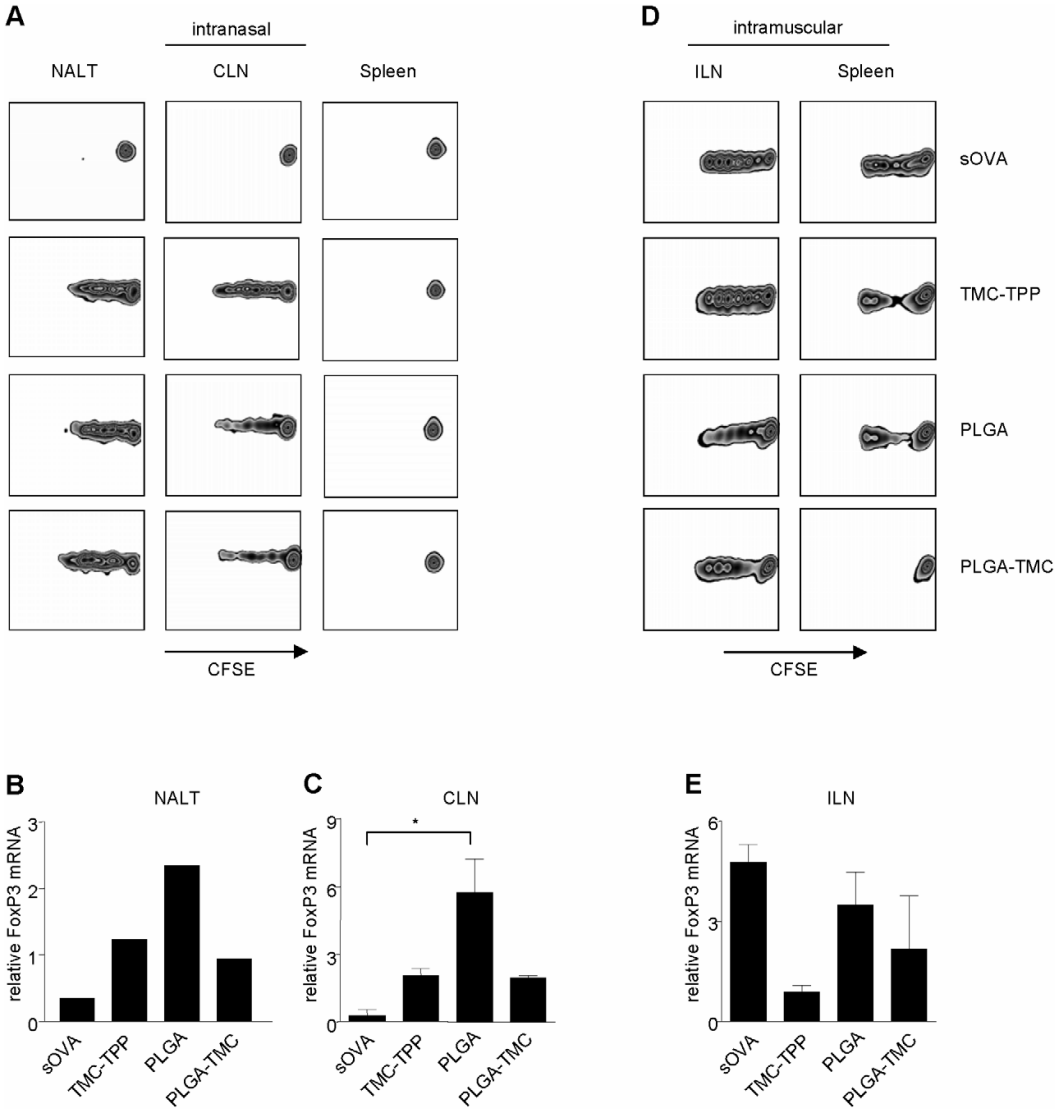
Enhanced OVA-specific CD4^+^ T-cell proliferation, after nanoparticle administration. **A and D.** OVA-specific CFSE labeled CD4^+^ T-cells were transferred to BALB/c recipient mice one day prior to vaccination. Mice received a single i.n. application of 30 µg of sOVA or OVA encapsulated into PLGA, PLGA-TMC or TMC-TPP nanoparticles. For induction of a non-mucosal response, mice received a single i.m. immunization in the hind limbs. At 72 h post OVA administration, *in vivo* T-cell division was addressed in spleen, nose-draining NALT and CLN as well as the thigh-draining ILN. Data are representative for at least 3 i.n. and 2 i.m. independent transfer studies. **B, C and E.** Total mRNA was purified from single cell suspensions from NALT, CLN, and ILN. Relative mRNA expression to HPRT of Foxp3 was determined 72 h post OVA application. Cells isolated from NALT were pooled per group. LN data are representative for at least 3 to 5 mice per group; mean ± SEM. Statistically significant: *, *P*<0.05.

First we studied the short-term CD4^+^ T-cell response in mice that were treated i.n. or i.m. with 30 µg of sOVA or OVA encapsulated particles. Proliferation of OVA-specific CFSE-labeled CD4^+^ T-cells was addressed locally in the draining lymph nodes as well as systemically in the spleen 72 h after treatment (Figures 2A and 2D).

Nasal vaccination induced strong local CD4^+^ T-cell proliferation in the nasopharynx-associated lymphoid tissue (NALT) and cervical lymph nodes (CLN), irrespective of the type of nanoparticle, whereas low-dose sOVA did not. None of the formulations induced measurable CD4^+^ T-cell activation in the spleen at 72 hours after vaccination upon i.n. immunization (Figure 2A). In contrast, non-mucosal vaccination resulted in proliferation both in the draining inguinal lymph nodes (ILN) and spleen at this time point (Figure 2D).

We did not detect significant differences in cytokine profiles in culture supernatants of the isolated draining CLN and ILN organs after particle vaccination (data not shown). However, we observed an increased expression in the relative Foxp3 mRNA expression in the CLN (Figure 2C) and a slightly increased expression in the NALT (Figure 2B) of mice that had received a single i.n. PLGA vaccination. Mice that were vaccinated i.m. with TMC-TPP particles showed less expression of Foxp3 mRNA compared to PLGA and PLGA-TMC treated mice in the ILN (Figure 2E).

These data show that i.n. vaccination with low-dose OVA encapsulated nanoparticles enhanced CD4^+^ T-cell proliferation in contrast to low-dose sOVA treatment (Figure 2A) and coincided with enhanced Foxp3 mRNA expression in the NALT and CLN only when PLGA encapsulated OVA was applied (Figures 2B and 2C). This effect was lacking in the i.m. treated mice of all treatment groups (Figure 2E) showing that, both particle and route of application determine the outcome of the CD4^+^ T-cell response.

### PLGA nanoparticle vaccination suppressed DTH response

**Figure 3:**
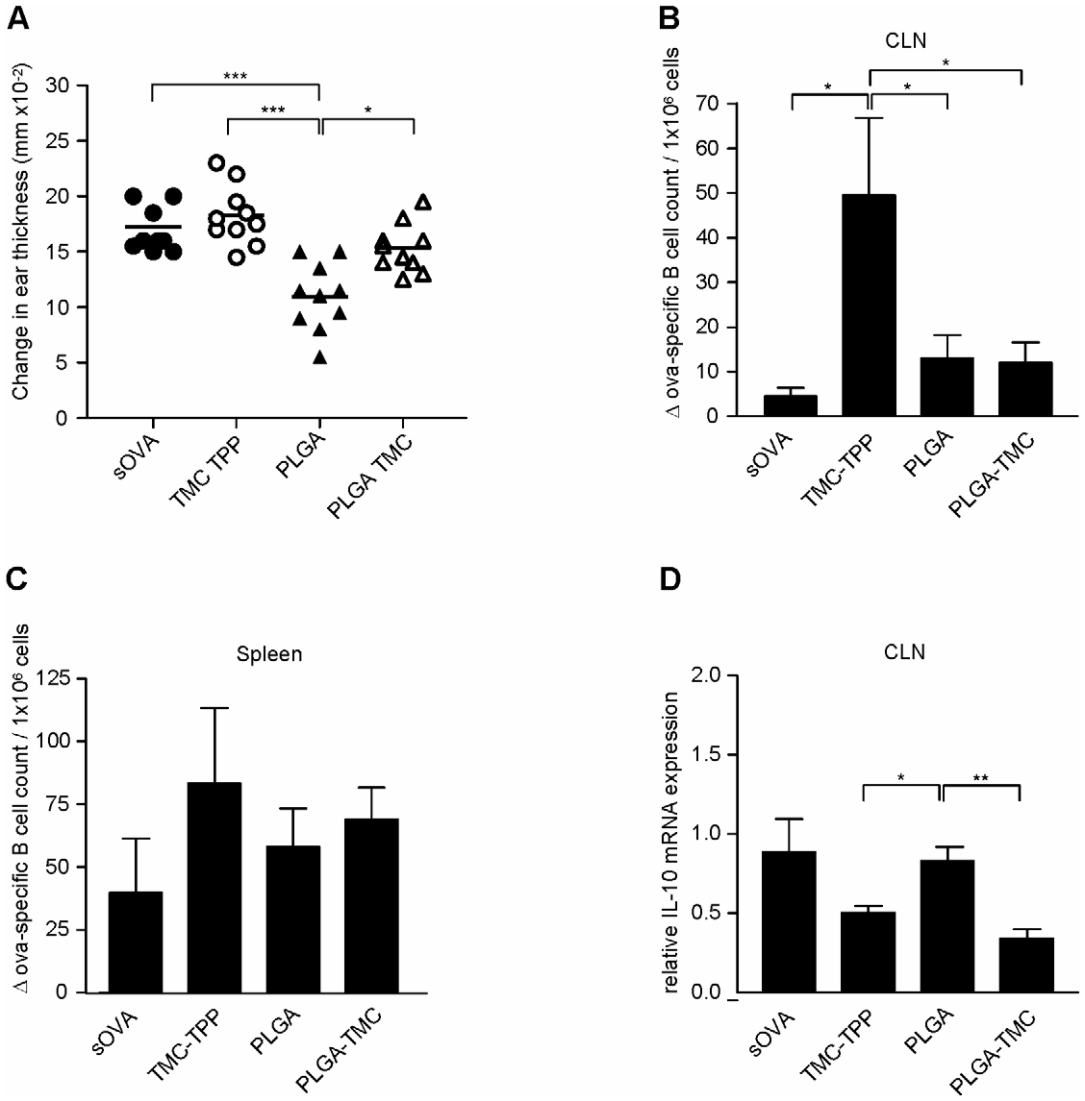
Nasal application of PLGA particles suppressed a Th-1-mediated hypersensitivity reaction, while TMC-TPP enhanced humoral immunity. **A.** Mice received 20 µg of OVA i.n. either dissolved in PBS (black circles) or encapsulated in TMC-TPP (white circles), PLGA (black triangles) or PLGA-TMC (white triangles) nanoparticles for three successive days. Mice were sensitized subcutaneously after nasal OVA administration and subsequently challenged in the auricle of both ears. Changes in ear-thickness were determined and compared with values before challenge. **B and C.** OVA-specific B-cell response induced after nasal nanoparticle treatment. OVA-specific B-cell response was assessed by ELISPOT. Data are shown as the OVA-specific B-cell count per 1*10^6^ cells from CLN and spleen above background (spots counted on medium coated plates). **D.** PLGA-TMC and TMC-TPP nanoparticles inhibit IL-10 mRNA expression in the CLN whereas PLGA induced IL-10 mRNA expression did not differ from sOVA. Relative mRNA expression to HPRT of IL-10 was determined in the CLN of mice. Data are shown as the mean ± SEM. of n = 5 mice per group. Statistically significant: *, *P*<0.05; **, *P*<0.01; ***, *P*<0.001.

To see if the differences in T-cell response induced after nasal treatment are of functional importance, the nanoparticles were tested for immunomodulation in a DTH-model. Mice received 20 µg of OVA i.n. three times at 24 h intervals either dissolved in PBS or encapsulated in PLGA, PLGA-TMC or TMC-TPP nanoparticles. Subsequently, mice were sensitized by OVA/IFA and challenged with OVA in the auricle of the ear. Ear-thickness was determined 24 h after challenge and compared with measures before challenge. PLGA nanoparticle treatment suppressed the OVA-specific DTH response, whereas PLGA-TMC and TMC-TPP nanoparticles did not (Figure 3A). Non-tolerized mice showed a strong ear-thickness response comparable to low dose sOVA (17.45±1.44), whereas mice tolerized by high dose sOVA significantly reduced a DTH response (9.1±1.21).

In contrast to PLGA and PLGA-TMC, nasal application of TMC-TPP led to a systemic OVA-specific B-cell response (Figure 3C) and significantly increased humoral immunity locally in the draining CLN (Figure 3B). In agreement with earlier studies, this suggested a role for TMC-TPP in the activation of the humoral immune response after nasal treatment [18]. In addition, PLGA-TMC and TMC-TPP nanoparticles seemed to have an inhibitory effect on IL-10 mRNA expression locally in the draining CLN, whereas PLGA induced IL-10 mRNA expression did not differ from sOVA (Figure 3D). Although we detected increased expression of relative Foxp3 mRNA in the T-cell transfer study (Figures 2B and 2C) we were not able to detect such differences in the DTH model, probably due to experimental differences in timing and the presence of OVA-specific T-cells in the transfer model.

To summarize, only nasal treatment with PLGA nanoparticles induced nasal tolerance to a low dose antigen but did not enhance humoral immunity as shown by the absence of antigen-specific B-cell responses (Figures 3A and 3B). In contrast to PLGA and PLGA-TMC immunization, only TMC-TPP treatment led to activation of humoral immunity as shown by local increased generation of antigen-specific B-cells (Figure 3B) and increased antigen-specific antibody titers systemically (see [18]).

### Enhanced protection against arthritis after nasal application of mB29a PLGA nanoparticles

**Figure 4:**
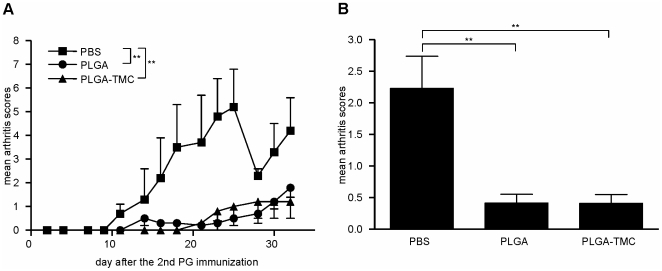
Nasal application of low-dose mB29a-PLGA containing particles reduces severity of arthritis. **A and B.** Effect of mB29a-nanoparticles on nasally induced suppression of PG-induced arthritis in BALB/c mice. Mice received 30 µg of mB29a peptide i.n. dissolved in PBS or encapsulated in PLGA or PLGA-TMC nanoparticles prior to arthritis induction. Arthritis scores of mB29a-PBS (black squares), PLGA (black circles) or PLGA-TMC (black triangles) treated mice as assessed by swelling and redness of the paws. Data are shown as the mean arthritis scores ± SEM. of n = 3 mice per group. Statistically significant: **, *P*<0.01.

We demonstrated that upon nasal application of OVA-PLGA nanoparticles, mice were able to suppress a local induced Th-1 mediated inflammatory response in an OVA-specific DTH-model. Additionally, we investigated whether enhanced nasal tolerance induction mediated by PLGA treatment was sufficient to suppress a chronic inflammatory response. Hsp70-peptide mB29a was encapsulated into each of the nanoparticles and we tested the capacity of the nanoparticles to modulate the response towards mucosal tolerance in the PGIA mouse model. Mice were tolerized by three times i.n. hsp70-peptide treatment either dissolved in PBS or encapsulated in nanoparticles prior to arthritis induction by two i.p. PG/DDA immunizations with a three week interval. These initial data showed proof of principle that treatment with PLGA nanoparticles reduced mean arthritis scores that lasted up to 30 days after disease development (Figures 4A and 4B), which correlated with earlier antigen release kinetics studies [18]. As expected, we did not observe an immunosuppressive function for TMC-TPP, as the mean arthritis scores (1.2±0.2) in these mice were not significantly lower than that of control mice (data not shown). Furthermore, the lower mean arthritis scores observed after PLGA or PLGA-TMC treatment coincided with a later onset of disease and lower maximum arthritis scores (Table 1).

**Table 1:** Onset of disease and maximum arthritis scores.

Treatment group	Onset of disease	Maximum arthritis scores
PBS	15.0±3.6	5.2±2.8
PLGA	23.0±8.2	1.2±0.6
PLGA-TMC	27.7±9.9	1.2±1.3

Hsp70-mB29a peptide loaded PLGA, PLGA-TMC nanoparticles or PBS control (10 µg) were given i.n. on day −7, −5 and −3 and arthritis was induced by PG/DDA immunization on day 0 and 21. Arthritis symptoms were scored as described in materials and methods. Day of onset and maximum arthritis scores were depicted as mean ± SEM. of n = 3 mice per group of one experiment.

## Discussion

In the area of vaccine development, nasal delivery is an attractive route also given the non-invasive needle-free administration [16], [32]. Earlier studies already showed that nasal nanoparticle treatment enhanced humoral immunity [4], [18] or suppressed this by mucosal tolerance induction [24], depending on the antigen-particle combination. Since there was not much known about the role of CD4^+^ T-cells upon nasal nanoparticle treatment, we explored how nanoparticle treatment affected CD4^+^ T-cell activation both *in vitro* and *in vivo*.

Particle characteristics modulated DC-induced OVA-specific CD4^+^ T-cell proliferation *in vitro* (Figure 1C) since low-dose sOVA was not able to activate T-cells whereas particle incorporated OVA had a differential capacity to do so. The proliferative response was not a result of antigen-independent particle induced activation since empty nanoparticles were not able to activate T-cells (data not shown). Although the uptake of sOVA-FITC in Figure 1A and 1B was more efficient in contrast to TMC-TPP nanoparticle treatment at various OVA concentrations, the amount of sOVA was insufficient to activate T-cells *in vitro* as shown in Figure 1C. In addition, sOVA-FITC uptake was similar to that of PLGA and PLGA-TMC at 1 µg/ml concentrations (Figure 1A and 1B) however it was not sufficient for sOVA to induce T-cell activation (Figure 1C). Although sOVA treated cells will also present their antigen via MHC class II we suggested that the expression of MHC molecules will be maintained for a shorter period of time in contrast to nanoparticle induced expression as previously described [33], [34]. Here, the authors showed that PLGA-microspheres *in vitro* induced prolongation of antigen presentation by the MHC class I molecule [33], [34] and MHC class II molecule [34] by antigen presenting cells.

Therefore, we suggest that nanoparticle mediated T-cell activation at similar low OVA concentrations was enhanced due to differences in MHC class II expression and antigen presentation in contrast to sOVA and that antigen uptake does not necessarily need to correlate with T-cell activation (Figure 1).

We showed that low-dose OVA-encapsulated nanoparticles enhanced OVA-specific CD4^+^ T-cell proliferation locally in the NALT and CLN after a single nasal application, which was not seen with low-dose sOVA. This showed the superiority of nanoparticle mediated OVA delivery versus sOVA delivery (Figure 2A). In addition, systemic CD4^+^ T-cell activation following nasal treatment requires a longer time frame as compared to non-mucosal antigen immunization (Figure 2). These data confirm that also the route of antigen delivery activates the immune system, as previously described [23].

Since CD4^+^CD25^+^Foxp3^+^ regulatory T cells play an important role in the induction of mucosal tolerance [35], [36], we explored Foxp3 expression after nasal nanoparticle treatment. Interestingly, Foxp3 mRNA expression was increased locally in the CLN, only after PLGA treatment (Figure 2C) although proliferation profiles were comparable for all particles (Figure 2A). This suggests that different nanoparticles induced a differential T-cell response and only T-cells activated in presence of PLGA nanoparticles obtained a tolerogenic phenotype as compared to nasal treatment with PLGA-TMC or TMC-TPP (Figures 2B and 2C).

And indeed nasal treatment with low-dose OVA-encapsulated PLGA nanoparticles induced a functional immunomodulatory T-cell response as shown by a reduced DTH response (Figure 3A). We did not observe a tolerogenic effect in the DTH model when mice were treated with low-dose sOVA alone. We suggest that this is due to the low-dose sOVA concentration (3×20 µg) that was used for tolerization of the mice since high-dose sOVA (3×100 µg) was sufficient to induce tolerance (Figure 3A) and ([23], [37]). The absence of stimulatory effects of the low-dose sOVA or OVA incorporated in the nanoparticles on dendritic cells argues against LPS contamination. Moreover, if our OVA contained high doses of LPS contamination, high-dose sOVA would not be expected to suppress a DTH.

Nevertheless, low-dose sOVA administration might still favor tolerance induction as shown by the IL-10 mRNA expression in the draining CLN (Figure 3D) however it might not be strong enough to actually suppress the inflammatory response.

Interestingly enough, PLGA particles that were capable of reducing DTH reactivity also had the capacity to induce IL-10 (Figure 3D), suggesting that the characteristics of the PLGA nanoparticle enhanced tolerance induction in contrast to low-dose sOVA.

No immunosuppressive reaction was seen in TMC-TPP treated mice that showed significantly increased OVA-specific B-cells in the CLN (Figure 3B) but not in spleen (Figure 3C). Although we were able to detect low titers of total IgG OVA-specific antibodies in the serum of treated mice, these differences were not significant (data not shown). We therefore concluded that at this time point no systemic but only a local OVA-specific B-cell response was induced in the CLN. In a previous study we explored the effect of nanoparticle treatment on B-cell activation during a 10-week vaccination study [18]. Here, nasal treatment with TMC-TPP nanoparticles resulted in significantly enhanced OVA-specific IgG titers in serum, in contrast to PLGA and PLGA-TMC treatment. Moreover, only after nasal vaccination with TMC-TPP nanoparticles, the antibody titers were comparable to those obtained after i.m. TMC-TPP treatment.

Taken together, these data suggest that upon nasal nanoparticle application, specifically TMC-TPP nanoparticles activate the humoral arm of the mucosal immune system (Figure 3B and [18]).

Additionally, we looked at nanoparticle-mediated immunomodulation after nasal application in a chronic inflammatory condition by using the PGIA model. In various experimental arthritis models, Hsp(-peptides) were shown to have a capacity to down modulate arthritis, which seems to be mediated by the induction of Hsp-specific regulatory CD4^+^ T-cells [38]–[41]. Recently, we uncovered a therapeutic potential for mouse Hsp70-peptide mB29a to suppress arthritis after nasal application [31].

Here we show that mB29a peptide encapsulation into PLGA and PLGA-TMC nanoparticles enhanced the tolerogenic capacity of the peptide. Intranasal treatment with 30 µg of peptide dissolved in PBS did not reduce the severity of arthritis compared to encapsulated peptide (Table 1). We have seen that nasal application of encapsulated antigen can induce a tolerogenic response not only in a typical Th1 mediated DTH response, but also in principle in a model of chronic and relapsing arthritis. A difference in tolerogenic capacity was observed for the PLGA-TMC particles that suppress PGIA completely (Figure 4), but only partially suppressed the OVA DTH response (Figure 3A). This difference can be the result of the nature of the antigen and the chronic nature of the model itself. Hsp70 in the PGIA model is a self antigen that is expressed also by immune cells of the host, thereby enhancing the regulatory T cells induced by the intranasal treatment. In addition, arthritis is a chronic inflammation in contrast to the DTH response prolonging the effective window of the tolerogenic effect of PLGA-TMC treatment.

In conclusion, our results indicate that nasal administration of antigen by PLGA-containing nanoparticles can enhance an immunosuppressive response even at a low antigen dose, while TMC-TPP nanoparticles enhance humoral immunity. As mentioned in the introduction both antigen and vaccine characteristic affect the induced type of the immune response. Our results confirm that particle and antigen combinations need to be carefully constructed to design successful vaccines that induce the preferred type of immune response.

These findings may help the development of nanoparticle based interventions to drive an antigen-specific immunomodulatory response and will enable future rational vaccine design for prophylactic and therapeutic vaccination, respectively in infectious diseases and autoimmune diseases.

## Methods

### Ethics statement

All mice were kept in our animal facility at the Central Animal Laboratory (GDL), Utrecht University, The Netherlands under standard housing conditions. Experiments were approved by the Animal Experiment Committee of the Utrecht University, Utrecht, The Netherlands (Permit Number: 2007.II.03.072 and 2009.II.08.075).

### Mice

Male BALB/c mice (8–12 weeks) and female BALB/c mice (retired breeders aged between 16–26 weeks) were purchased from Charles River Laboratories (Maastricht, The Netherlands). OVA-specific TCR transgenic (Tg) mice on BALB/c background (DO11.10 mice), were bred at the Central Animal Laboratory (GDL), Utrecht University, The Netherlands.

### Antibodies and antigen encapsulated nanoparticles

In all *in vitro* and *in vivo* experiments, endotoxin-low OVA was purchased at Calbiochem (San Diego, CA). Murine Hsp70 peptide mB29a (HspA9-derived sequence VLRVINEPTAAALAY) was encapsulated into PLGA, PLGA-TMC or TMC-TPP nanoparticles and OVA-encapsulated PLGA, PLGA-TMC and TMC-TPP nanoparticles were generated as described previously [17], [18]. For detailed information about the particle characteristics such as size, zeta potential, loading efficiency and the polydispersity index, we refer to the paper published by Slütter et al. [18]. Anti-DO11.10 TCR (KJ1.26) was purchased from Molecular Probes (Invitrogen, Breda, The Netherlands), 7-Amino-actinomycin-D (7-AAD)-unconjugated, Anti-CD11c (HL3), anti-CD4 (RM4-5), anti-CD40 (3/23), anti-CD86 (GL1), anti-MHC class II (M5/114), antibodies were purchased from BD Pharmingen (Woerden, The Netherlands). Anti-Foxp3-PE (FJK-16s) and an appropriate isotype control were purchased from eBioscience (Breda, The Netherlands)..

### DC culture

Bone marrow-derived dendritic cells (BMDC) were cultured from BALB/c donor mice as previously described with minor modifications [42]. Briefly, femurs and tibia of adult BALB/c mice were flushed with culture medium. Single cell suspensions were seeded in complete Iscove's Modified Dulbecco's Medium (IMDM) supplemented with 5×10^−5^ M 2-mercaptoethanol, penicillin (100 units/ml) and streptomycin (100 µg/ml) (Gibco, Karlsrule, Germany) and 20 ng/ml murine rGM-CSF (Cytogen, The Netherlands). On day 2 and 4, 10 ng/ml murine rGM-CSF was added. The cells were cultured in a humidified 5% CO_2_ atmosphere at 37°C. On day 7, the BMDCs were routinely pure between 70% and 80% based on CD11c and MHC class II expression and used for further experiments.

### CD4^+^ T- cell enrichment and CFSE labeling

Spleens were isolated from DO11.10 donor mice and were prepared into single cell suspensions. CD4^+^ T-cells were obtained by negative selection with sheep-anti-rat IgG Dynabeads (Dynal, Invitrogen, Breda, The Netherlands) using an excess amount of anti-B220 (RA3-6B2), anti-CD11b (M1/70), anti-MHC class II (M5/114), anti-CD8 (YTS169) mAb. Enriched CD4^+^ T-cells were routinely pure between 85 and 90%. Labeling of cells with carboxy-fluorescein diacetate succinimidyl ester (CFSE; Molecular Probes, Leiden, The Netherlands) was performed as previously described [43].

### *In vitro* effect of nanoparticles on DC

To address maturation of BMDC by nanoparticles, BMDC were cultured in the presence of PLGA, PLGA-TMC or TMC-TPP nanoparticles containing 25 ng/ml to 1.0 µg/ml OVA or 10 ng/ml LPS (Sigma) as a maturation control. After 24 h, DC maturation was determined by flow cytometry (FACS-Calibur; BD Pharmingen) and FlowJo Software V8.8.6.

BMDCs were incubated for 1.5 hours at either 4°C or 37°C with FITC-labeled OVA protein purchased from Molecular probes (Invitrogen, Breda, The Netherlands) dissolved in PBS or with antigen incorporated into PLGA, PLGA-TMC or TMC-TPP. To quench external FITC, trypan blue stain (Gibco, Invitrogen) was added to each sample 5 minutes before FACS analysis at a final concentration of 0.02% and uptake was analyzed by flow cytometry. BMDCs were pre-incubated at 37°C for 2 h in the presence of OVA protein dissolved in PBS or incorporated in nanoparticles (PLGA, PLGA-TMC or TMC-TPP) at concentrations of 25 ng/ml, 0.5 µg/ml or 1.0 µg/ml. OVA-specific CD4^+^ T-cells were added at an 1∶10 DC∶T-cell ratio and T-cell proliferation was assessed after 72 h by CFSE dilution.

### T-cell activation in the local lymph nodes after nanoparticle vaccination

BALB/c recipient mice were adoptively transferred with 1.10^7^ CFSE-labeled CD4^+^KJ1.26^+^ cells in 100 µl PBS, intravenously. The next day mice received a single application of 30 µg of OVA dissolved in 10 µl of PBS or encapsulated into PLGA, PLGA-TMC or TMC-TPP nanoparticles i.n. or intramuscular i.m. in the hind limbs. 72 h after i.n. or i.m. OVA administration, the spleen, NALT and CLN as well as the thigh-draining ILN were harvested and single cell suspensions were analyzed.

### Delayed-type hypersensitivity (DTH) reaction

BALB/c mice received 20 µg of OVA i.n. three times at 24 h intervals either dissolved in PBS or encapsulated in PLGA, PLGA-TMC or TMC-TPP nanoparticles. Control groups received PBS alone or OVA at a final concentration of 100 µg in PBS. Mice were sensitized for a DTH the next day with 100 µg of OVA in 25 µl of PBS, mixed with 25 µl of Incomplete Freund's Adjuvant (IFA) (Difco, BD. Alphen a/d Rijn, The Netherlands) subcutaneously (s.c.) administered in the tail base. Five days later, ear-thickness of both ears was measured with an engineer's micrometer (Mitutoyo, Tokio, Japan). Subsequently, mice were challenged with 10 µg of OVA in 10 µl of PBS given in the auricle of each ear and 24 h post-challenge, the increase in ear thickness of both ears was determined.

The *early* B-cell response was assessed by detection of OVA-specific B-cells of immunized mice by ELISPOT. Single cell suspensions from the NALT, CLN and spleen were cultured with OVA (1 µg/well) or control on high protein binding filter plates (MultiScreen-IP, Millipore) for 48 hours. After incubation, spot forming units were detected with goat-anti mouse IgG-biotin (Sigma) and Avidin-AP (Sigma). Plates were developed with NBT-BCIP (Roche) and analyzed by using the Aelvis spotreader and software. Data are shown as the OVA-specific B-cell count per 1*10^6^ cells (antigen-induced-background).

### Nasal tolerance induction and assessment of arthritis

Female retired breeder BALB/c mice were treated 3 times with 10 µg of mouse Hsp70- peptide mB29a in PBS or encapsulated into PLGA, PLGA-TMC or TMC-TPP nanoparticles i.n. dissolved in 10 µl PBS on days −7, −5, −3. Arthritis was induced by intraperitoneal (i.p.) injections of 300 µg proteoglycan (PG) protein with 2 mg of the synthetic adjuvant dimethyl-dioctadecyl-ammoniumbromide (DDA) (Sigma) emulsified in PBS (total volume of 200 µl) on day 0 and day 21 as described [44], [45]. After the second PG immunization, the onset and severity of arthritis were determined using a standard visual scoring system based on swelling and redness of the paws as described [44].

### Luminex

CD4^+^ T-cells isolated from the spleen of DO11.10 mice were incubated with BMDCs isolated from BALB/c wt mice at an 1∶10 DC∶T-cell ratio and stimulated with sOVA or OVA encapsulated in nanoparticles in complete IMDM supplemented with 5×10^−5^ M 2-mercaptoethanol, penicillin (100 units/ml) and streptomycin (100 µg/ml) (Gibco, Karlsrule, Germany) at 37°C. The amount of cytokine secreted after a 72 h T-cell re-stimulation was assessed by analyzing the culture supernatants. Briefly, fluoresceinated microbeads coated with capture antibodies for simultaneous detection of IFN-γ (AN18), IL-2 (JES6-1A12), IL-10 (JES5-2A5), (BD Biosciences Pharmingen) were added to 50 µl of culture supernatant. Cytokines were detected by biotinylated antibodies IFN-γ (XMG1.2), IL-2 (JES6-5H4), IL-10 (SXC-1), and PE-labeled streptavidin (BD Biosciences Pharmingen). Fluorescence was measured using a Luminex model 100 XYP (Luminex, Austin, TX, USA).

### RT-PCR analysis

Total mRNA was purified from single cell suspensions from NALT, CLN, ILN or spleen using the RNeasy kit (Qiagen Benelux B.V.) according to the manufacturer's protocol. RNA was reverse transcribed into cDNA using the iScript™ cDNA Synthesis Kit (Bio-Rad Laboratories, B.V) according to the manufacturer's protocol. RT-PCR was performed using a MyiQ Single-Color RT-PCR detection system (Bio-Rad Laboratories B.V.) based on specific primers and general fluorescence detection with SYBR Green (iQ SYBR Green Supermix, Bio-Rad laboratories, Hercules, CA). Conditions for the Real-time quantitative reaction were (95°C for 3 min and 40 cycles of 95°C for 10 s and 59.5°C for 45 s). Expression was normalized to the detected Ct values of hypoxanthine-guanine phosphoribosyltransferase (HPRT) for each sample. The expression levels relative to HPRT were calculated by the equation: relative expression level = 2^−ΔΔCt^ (Livak Method). Specific primers were designed across different constant region exons resulting in the following primers:

**HPRT** sense 5′-CTGGTGAAAAGGACCTCTCG-3′, antisense 5′-TGAAGTACTCATTATAGTCAAGGGCA-3′. **IL-10** sense 5′- GGTTGCCAAGCCTTATCGGA-3′, antisense 5′-ACCTGCTCCACTGCCTTGCT-3′. **Foxp3** sense 5′-CCCAGGAAAGACAGCAACCTT-3′, antisense 5′-TTCT CACAACCAGGCCACTTG-3′.

### Statistics

Statistical analysis was performed with Prism software (Graphpad Software Inc., San Diego, version 4.00) using a one-way ANOVA followed by a Kruskal-Wallis test and Dunn's multiple comparison test was used for the *in vitro* cytokine and FoxP3 mRNA assay and a one-way ANOVA followed by Bonferroni's multiple comparison test was used for statistical analysis of the Delayed-type hypersensitivity reaction. An unpaired two-tailed Student's *t* test was used for statistical analysis in all other experiments. Error bars represent the S.D. or S.E.M.. as indicated. Statistical differences for the mean values are indicated as follows: *, *P*<0.05; **, *P*<0.01; ***, *P*<0.001.
